# Development and Field Evaluation of Near-Isogenic Lines of GR2-EBRRI dhan29 Golden Rice

**DOI:** 10.3389/fpls.2021.619739

**Published:** 2021-02-25

**Authors:** Partha S. Biswas, B. P. Mallikarjuna Swamy, Md. Abdul Kader, Md. Alamgir Hossain, Raul Boncodin, Mercy Samia, Md. Lutful Hassan, M. Wazuddin, Donald MacKenzie, Russell Reinke

**Affiliations:** ^1^Plant Breeding Division, Bangladesh Rice Research Institute, Gazipur, Bangladesh; ^2^International Rice Research Institute, Los Baños, Philippines; ^3^Department of Genetics and Plant Breeding, Bangladesh Agricultural University, Mymensingh, Bangladesh; ^4^Donald Danforth Plant Science Center, Saint Louis, MO, United States

**Keywords:** golden rice, vitamin A deficiency, provitamin A, marker-assisted backcrossing, confined field trial, *Indica* rice

## Abstract

Vitamin A deficiency remains a common public health problem among the rice-dependent poor people in the developing countries of Asia. Conventional milled rice does not contain provitamin A (β-carotene) in is edible part (endosperm) and is also deficient in essential minerals, such as iron and zinc. Transgenic Golden Rice event GR2E, which produces β-carotene in its endosperm, was used as a parent to introgress the transgene locus conferring β-carotene biosynthesis into a widely grown rice variety, BRRI dhan29, which covers around 26.1% of the irrigated rice area (4.901 Mha) of Bangladesh in the dry season. The current study reports the introgression process and field performance of GR2E BRRI dhan29 Golden Rice. The background recovery of GR2E BRRI dhan29 lines at BC_5_F_2_ generation was more than 98% with a 6K SNP-chip set. The transgenic GR2E BRRI dhan29 yielded 6.2 t/ha to 7.7 t/ha with an average of 7.0 ± 0.38 t/ha, while the non-transgenic BRRI dhan29 yielded 7.0 t/ha under confined field conditions in Bangladesh. Moreover, no significant difference between GR2-E BRRI dhan29 Golden Rice and non-transgenic BRRI dhan29 in any measured trait was observed in the multi-location trials conducted at five locations across the country. Furthermore, the appearance of cooked and uncooked rice was similar to that of BRRI dhan29 except for the yellow color indicating the presence of carotenoids. Total carotenoid content in the selected introgression lines ranged from 8.5 to 12.5 μg/g with an average of 10.6 ± 1.16 μg/g. This amount is sufficient to deliver approximately 66 and 80% of the recommended daily intake of vitamin A for children and women, respectively, assuming complete substitution of white rice in the diet with Golden Rice. However, the lead selected line(s) need further evaluation at open field conditions before deciding for commercial cultivation. A large-scale feeding trial among the malnourished community with this newly developed GR2-E BRRI dhan29 Golden Rice is also required to validate its efficacy in alleviating vitamin A deficiency.

## Introduction

Vitamin A deficiency (VAD) is a common public health problem among children and pregnant women in the developing countries of Asia. The highest prevalence of VAD, which affects about one-third of the children aged 6–59 months worldwide, was observed in sub–Saharan Africa (48%) followed by South Asian (44%) countries (Stevens et al., 2015). Prevalence of subclinical VAD among preschool-aged children in these countries was estimated to range between 21.7 and 62.0%. VAD is widespread and persistent in Asian countries where rice is the only food for many people for energy, minerals, and vitamins. In most of the South Asian countries, 70–80% of the main course of the diet of common poor people comes from rice; very small portion comes from other sources. However, the precursor to vitamin A, β-carotene, does not exist in the edible part of natural rice germplasm (Bouis, 2000; Beyer, 2010). Mortality associated with malnutrition and higher prevalence of VAD among neonatal and children aged below 5 years in Bangladesh and India constituted 30% of the global mortality rates (World Health Organization, 2009). Bangladesh has persistent VAD, despite ongoing vitamin A supplementation programs, food fortification, motivational activities for dietary diversification, and campaigns for optimal breastfeeding for infants. Biofortification of staple food crops has already shown promise as a sustainable approach to alleviating micronutrient malnutrition in many countries in Asia and Africa (Bouis and Saltzman, 2017). Transgenic Golden Rice (Paine et al., 2005), an example of biofortification, in which endosperm-specific biosynthetic pathways of provitamin A were introduced through genetic modification taking *psy* gene from maize (*Zea mays*) and *crtI gene* from a common soil bacteria, *Pantoea ananatis* (syn. *Erwinia uredovora*), is a complementary approach to help sustainably combat VAD. However, the Golden Rice events were developed in a tropical *japonica* genetic background (Paine et al., 2005), and this background is not suitable for direct use in commercial cultivation due to poor adaptation to the more tropical environment of Bangladesh and less consumer preference for sticky cooked rice (Virk et al., 2006). The Golden Rice events developed earlier by Datta et al. (2003) and Baisakh et al. (2006) in different *indica* backgrounds showed relatively low carotenoid content (up to 9.34 μg/g) (Datta et al., 2007). The Golden Rice events (Paine et al., 2005) developed in the background of US long-grain tropical *japonica* variety Kaybonnet had β-carotene levels as high as 37 μg/g in the uncooked rice. The latter studies showed that a substantial amount of β-carotene retains (∼60%) even after cooking. After rigorous review at the International Rice Research Institute (IRRI), the GR2E event was selected as the donor for conversion of the elite *indica* rice varieties grown in South and Southeast Asian countries (Humanitarian Board, 2009; Beyer, 2010; Dubock, 2014, 2019).

Backcrossing is a simple breeding technique to transfer a trait of interest from a donor into elite varieties. With the advent of molecular marker technology, the process has become faster and more efficient in avoiding undesired linkage drag. Marker-assisted backcrossing (MABC) was used successfully in the introgression process of different traits into several mega varieties of rice [e.g., Sanchez et al. (2000) for bacterial leaf blight resistance, Neeraja et al. (2007) and Iftekharuddaula et al. (2015) for submergence tolerance; Zhou et al. (2003) for grain quality traits]. In this study, we used MABC to introgress the transgene locus from *japonica* Kaybonnet GR2E Golden Rice into an *indica* variety, BRRI dhan29. Rice is widely grown and consumed in Bangladesh and some rice varieties like BR11, BRRI dhan28, and BRRI dhan29 are very popular among the farmers. BRRI dhan29 alone covers more than 26.1% of the rice areas (ca. 4.901 Mha) under irrigated ecosystem (*Boro*) in Bangladesh (Bangladesh Rice Research Institute, 2019; Bangladesh Bureau of Statistics, 2020) and contributes a big share to the total production. It yields 7–8 t/ha under optimum management conditions in the *Boro* season (November to May). Since introgressed versions of the popular varieties developed through MABC usually do not differ significantly in appearance except for the introgressed trait, they are easily accepted by the farmers. Thus, it is expected that the Golden Rice version of BRRI dhan29 would more readily reach those in need and at risk of VAD. Therefore, this study was undertaken to introgress the GR2E locus into BRRI dhan29 to develop near-isogenic lines (NILs) of GR2E BRRI dhan29 Golden Rice and to identify superior lines after necessary evaluation under confined field conditions for further advancement.

## Materials and Methods

This study was conducted in two steps—firstly, development of NILs of GR2E BRRI dhan29 Golden Rice and secondly, evaluation of the NILs under confined conditions. The introgression work was done at IRRI, Philippines during 2008–2014. The selected BC_5_F_3_ lines were transferred to Bangladesh in 2015 and evaluated under confinement in successive years in different locations in Bangladesh.

### Plant Materials

**FIGURE 1:**
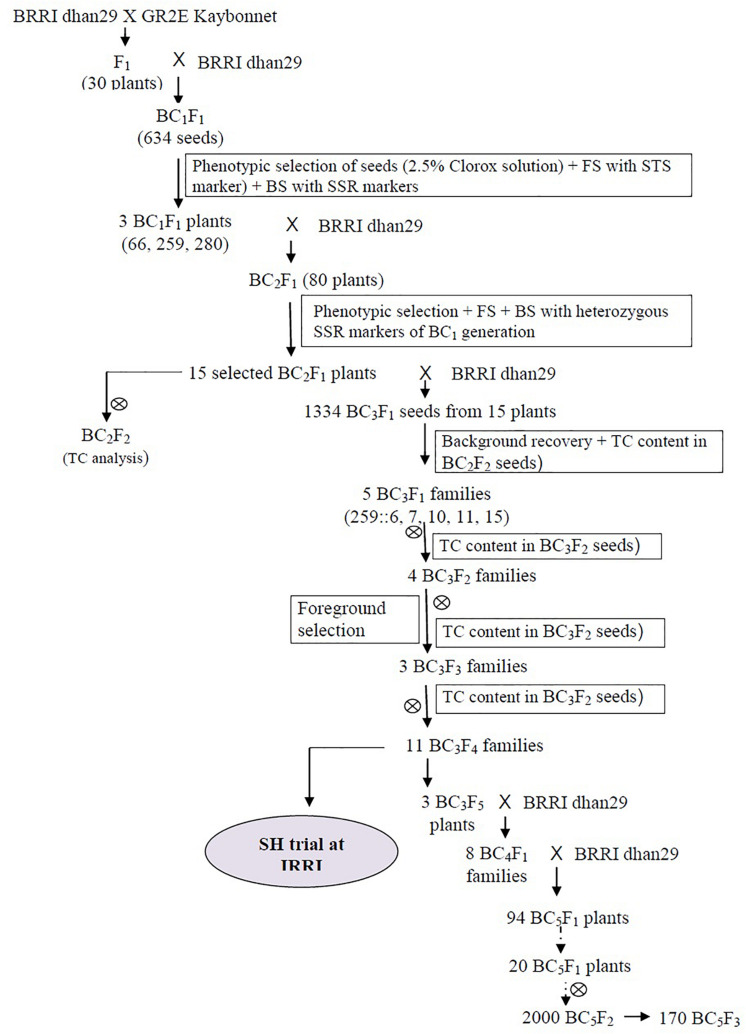
Breeding scheme for the development of the GR2E BRRI dhan29 Golden Rice near-isogenic lines. FS, BS, BC, and TC stand for foreground selection, background selection, backcross, and total carotenoids, respectively.

Kaybonnet GR2E Golden Rice (hereafter GR2E Golden Rice) was used as the donor parent for transgenes and BRRI dhan29 was used as the recipient to make crosses for this study at the IRRI, Philippines. The transgenic GR2E Golden Rice had an intact, single-copy transgene insert (Paine et al., 2005; Trijatmiko et al., 2015; Oliva et al., 2020) on chromosome 3. In our previous study (unpublished) conducted in 2008, the total carotenoids (TCs) and β-carotene levels of the GR2E event were 21.5 μg/g and 13.3 μg/g, respectively, in the unparboiled polished grains of fresh harvest samples from the screenhouse (SH) at IRRI. However, the carotenoid levels were found to stabilize after 7–8 weeks and remained almost static. On the other hand, BRRI dhan29, a high-yielding rice variety, yields 7–8 t/ha under optimum management conditions in the *Boro* season (November to May) in Bangladesh. BRRI dhan29 was crossed with GR2E Golden Rice (IR-∅∅GR2E-5), and the resulting F_1_ progenies were crossed further with BRRI dhan29 as a recurrent parent to generate backcross generations up to the BC_5_ generation (Figure 1). Self-pollinated seeds of different generations were used to analyze TC content in the polished grains.

### Phenotypic Selection of Seeds

In Golden Rice, expression of the transgene construct results in characteristically yellow grain endosperm due to accumulation of β-carotene but the color of the endosperm of F_1_ or BC_*n*_F_1_ seed is not visible due to its outer thick brown aleurone layer. Before sowing, all F_1_ or BC_*n*_F_1_ seeds were treated with 2.5% commercial bleach (NaOCl) solution in 50 ml falcon tubes at a rate of 25 ml per 100 seeds and were shaken on an orbital shaker at 100 rpm for 10 min to remove the outer brown layer (Biswas, 2011). The bleaching solution was discarded, and the seeds were rinsed with distilled water four to five times before placing in the Petri dishes for germination. The seeds with yellow colored kernels were used for germination, while seeds with white kernel were discarded from the study.

### Molecular Marker Analysis

Genotyping was performed with GR2E event-specific simple tagged sequence (STS) marker to track the transgene. Background recovery of the recurrent parental genome at early backcross generations was monitored using simple sequence repeats (SSR) markers. Single-nucleotide polymorphism (SNP) markers were used to confirm background recovery in the advanced backcross progenies. DNA was extracted from fresh and young leaf tissue following a modified CTAB method (Murray and Thompson, 1980). PCR was performed in 10 μl reactions containing 25 ng of DNA template, 1 μl of 10 × PCR buffer (containing 200 mM Tris–HCl, pH 8.3, 500 mM KCl, and 15 mM MgCl_2_), 1 μl of 1 μM dNTPs, 0.5 μl each of 5 μM forward and reverse primers (in case of STS, a third gene-specific primer was added), 0.3 μl of 20 mM MgCl_2_ (in case of STS analysis), and 0.2 μl of Taq DNA polymerase (5 U/μl) using a G-storm thermal cycler with initial denaturation temperature 94°C (95°C for STS) for 5 min followed by 45 s denaturation at 94°C (95°C for STS), 45 s annealing at 55°C, and 1.5 min extension at 72°C in each cycle with a final extension for 7 min at 72°C at the end of 35 cycles. The PCR product was separated by electrophoresis in polyacrylamide gel and agarose gel (the latter was used to separate STS and SSR markers with higher molecular weight). SSR markers on the rice linkage map of Temnykh et al. (2001) were selected based on information from McCouch et al. (2002) and International Rice Genome Sequencing Project (2005) considering the sequential order as physical distance. An SSR-polymorphism survey between BRRI dhan29 and GR2E was conducted with 239 SSR markers distributed over the 12 chromosomes of rice. A set of 104 polymorphic SSR markers was used for background selection. The SSR markers fixed with the recipient alleles (homozygous) were not analyzed in the succeeding backcross generations. The fixed lines at BC_3_F_4_ generations were analyzed with 384-plex SNP set using Illumina Golden Gate genotyping technology (Steemers and Gunderson, 2005; Illumina, 2006), while in BC_4_ and BC_5_ generations 6K SNP chip set (Thomson et al., 2017) was used for confirmation of background recovery.

### Analysis of Carotenoid Contents

Carotenoids are a group of plant pigments of which β-carotene and some other variants such as beta-cryptoxanthin are converted in the body into vitamin A on an “as needed” basis. A very high proportion (80–90%) of TCs in Golden Rice is β-carotene (Paine et al., 2005). Thus, TC concentration, which is easily measured spectrophotometrically after hexane extraction, was used as a major selection criterion along with genotyping and phenotypic comparison with their recurrent parent. At every backcross generation, two panicles from each plant used in backcrossing were allowed to produce self-pollinated seeds. TCs were extracted from polished grains of self-pollinated seeds after 2 months of storage in a brown paper bag at ambient temperature following the protocol described in Paine et al. (2005), with slight modification. Lipophilic metalloorganic dye VIS682A (QCR Solutions Corporation) was used as an internal standard to determine the correction factor for recovery of the extracts. TC content was estimated by measuring the extract’s absorbance at 450 nm using an UV/Vis spectrophotometer (DU^®^ 730, Beckman Coulter) and calculated applying Beer–Lambert’s Law according to Goodwin (1976) and Rodriguez-Amaya and Kimura (2004). The recovery of carotenoids content was corrected with a recovery factor derived from the internal standard read at 680 nm.

### Evaluation of Agronomic Performance

The agronomic performance of GR2E BRRI dhan29 NILs was evaluated under contained SH conditions in the Philippines and Bangladesh, and in confined field trials (CFTs) in Bangladesh. The first contained SH trial was conducted at IRRI, Los Baños, Philippines, during the dry season in 2010. The second contained SH trial was conducted at the Bangladesh Rice Research Institute (BRRI), Gazipur, Bangladesh, in 2015 during Aus season (April to September). The first CFT was conducted at BRRI, Gazipur (23°59′12″N and 90°24′0.50″E) in the Boro season (November to May) of 2016. Selected materials from the CFT were tested at five locations across the country with geographical coordinates, 23°59′12″N, 90°24′0.50″E; 22°40′53.4″N, 90°21′31.8″E; 24°22′16.7″N, 88°39′37.9″E; 23°28′08.7″N, 91°09′34.1″E, and 24°25′03.8″N, 91°25′39.7″E during the Boro season of 2017 and 2018. Average agroclimatic conditions (temperature and rainfall profiles) during the trial period of the CFT sites can be seen in Supplementary Figure 1.

In the SH trials at IRRI, 11 BC_3_F_4_ NILs along the recipient parent BRRI dhan29 were evaluated in a randomized complete block design with two replications. Twenty-one-day old seedlings of each parent/line were transplanted into 2.6 m × 3-row plots with rows spaced at 20 cm and hills in rows at 20 cm. In case of the SH trial at BRRI, 25-day-old single seedlings of 170 BC_5_F_3_ NILs along with wild-type BRRI dhan29 as check variety were transplanted at a spacing of 25 cm × 20 cm in 2.75–3.0 m-long three-row plots, while in the CFT, 23 BC_5_F_4_ NILs along with recipient parent BRRI dhan29 as check variety were tested in a randomized complete block design with three replications. Thirty-five-day-old seedlings of each NIL were planted in 5.0 m × 1.0 m plots with rows spaced at 20 cm and hills in rows at 20 cm. The number of hills per plot was 125 with one seedling transplanted per hill. Three border rows of wild-type BRRI dhan29 were transplanted surrounding each block. Standard crop management practices were followed for conducting the trials. Throughout the growth period of the trials, the plants were observed for visual similarity with BRRI dhan29 in different morpho-agronomic traits, and disease and insect–pest reaction. Avoiding border rows, 10 random plants were taken to record observations of plant height, panicle length, flag leaf length, flag leaf breadth, numbers of spikelets/panicle, spikelet sterility (%), numbers of panicles/hill, and grain yield/plant at 14% moisture. Days to maturity was recorded on whole plot basis. In the CFT, grain yield was recorded from the whole plot after processing and drying of seeds and converted to t/ha at 14% moisture. Grain length and width were recorded from 25 randomly selected, fully filled grains and thousand-grain weight was estimated from the weight of 500 grains adjusted at 14% moisture. Hundred-grain weight was recorded from 100 grains at 14% moisture in case of multi-location CFTs. Amylose content and TCs were measured from polished grains of fully ripened grains. Observation on reaction to diseases and insect pests were recorded under field conditions through regular monitoring.

### Data Analysis

Graphical genotype (GGT 2.0; Van-Berloo, 1999) and Flapjack (Milne et al., 2010) analytical tools were used to estimate background recovery for SSR and SNP genotyping data, respectively. The chi-squared (χ^2^) test for goodness of fit was used to estimate the segregation ratios in each backcross generation. Recipient parent genome (RPG) in the backcross progenies was calculated in percentage following the formula, RPG = [(X + 1/2Y) × 100]/N; where, “X” is the total number of markers homozygous for recipient parent allele, “Y” is the total number of markers heterozygous alleles, and “N” stands for the total number of polymorphic markers used in background genotyping. Agronomic data were analyzed using a linear mixed model in R package Agricolae v1.3-2. Student’s *t*-test was conducted between the mean performances of the NILs and the recipient parent (BRRI dhan29) for different agronomic traits to investigate genetic similarity.

## Results

### Development of Introgression Line

#### Progeny Selection for the Presence of GR2E Locus

**FIGURE 2:**
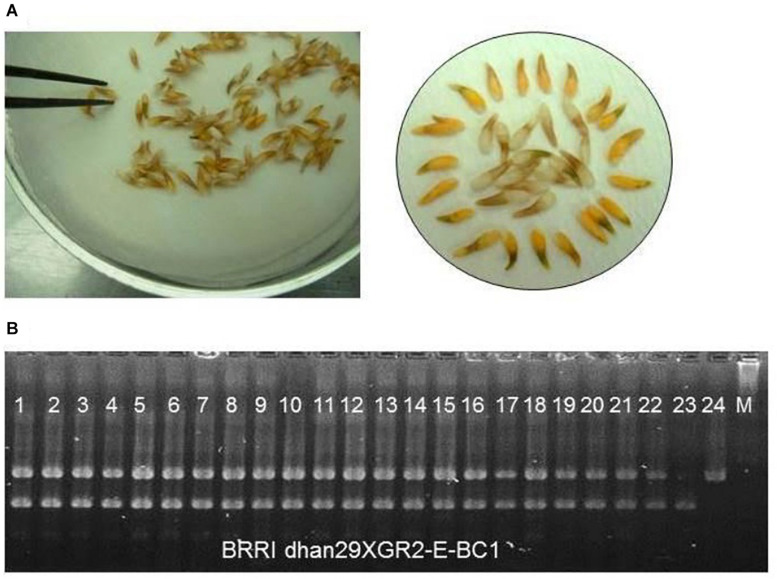
Selection of progenies for transgene GR2E locus. **(A)** Screening for presence of the transgene by visual inspection for yellow-colored seeds using a 2.5% NaOCl solution to remove the aleurone layer. Seed showing yellow kernel represents the presence of transgene and seed having white kernel represents vice versa. **(B)** A partial gel image of foreground genotyping with GR2E event-specific STS markers showing hemizygous loci for transgene (Lanes 1–22). Lanes 23 and 24 represent donor parent GR2E Kaybonnet and recipient BRRI dhan29, respectively.

Phenotypic selection of seeds with a yellow kernel and foreground selection with event-specific STS marker were used to track the GR2E locus in the backcross progenies (Figure 2). In the BC_1_F_1_ generation, 428 seeds out of 634 were found to have yellow kernel after bleaching with 2.5% NaOCl, and all the plants (428) derived from those seeds were hemizygous GR2E locus in foreground selection. The chi-squared test between the progenies with and without the GR2E locus showed a non-significant value (0.763) at *P* = 0.05 and fitted the expected 1:1 ratio in this generation. In the BC_2_F_1_ generation, 80 plants were isolated as hemizygous for GR2E locus from 176 plants, while 123 plants grown from 235 seeds were found hemizygous for the GR2E locus in the BC_3_F_1_ generation. The chi-squared values were 1.45 and 0.51, respectively, in the BC_2_F_1_ and BC_3_F_1_ generations. These values also perfectly fitted the expected 1:1 ratios between the individuals with and without transgene locus. The transgene locus was fixed at BC_3_F_2_ by selfing of BC_3_F_1_ plants. Eight BC_3_F_2_ plants were found to be homozygous for transgene locus out of 36 plants used in the foreground selection. At BC_3_F_3_ generation, 100 seeds of each of the selected three plants were dehulled and bleached to confirm the zygosity of the transgene GR2E. All kernels in all plants were yellow, thus confirming the homozygosity of the transgene locus. Table 1 summarizes the number of backcross-derived lines used in different selection methods at different generations for progeny selection.

**TABLE 1:** The number of plants used for generation advancement and recovery of the recurrent parent genotype at different backcross generations of GR2E BRRI dhan29.

Generation	No. of seeds used in bleaching	No. of plants used in foreground selection	No. of plants used in background selection	No. of plants used in backcrossing/TC analysis	No. of plants selected for next cycle	% RPG of the selected plants*
BC_1_F_1_	634	428	233	–	3	83.2–84.2
BC_2_F_1_	176	80	63 (13 + 15 + 35)	15	5	84.8–88.0
BC_3_F_1_	235	123	45 (8 + 11 + 9 + 4 + 13)	12	3	88.9–90.5
……	–	–	–	–	–	–
BC_3_F_4_	–	–	22	11	3	92.5–96.2
BC_4_F_1_	–	–	94	11	8	>86
BC_5_F_1_	–	–	94	94	20	>98

*The recovery of RPG values of the selected plants from BC_1_F_1_, BC_2_F_1_, and BC_3_F_1_ were based on 104 SSR marker data, that from BC_3_F_4_ generations was based on Illumina 384 SNP chip set, and those from BC_4_F_1_ and BC_5_F_1_ generations were based on 6K SNP chip set.

#### Progeny Selection for Phenotypic Similarity

Before background genotyping at each generation, the plants were phenotypically characterized based on visual observation of plant height, tillering pattern, and compactness of the canopy, erectness of flag leaf, flag leaf shape and size, and flowering time. The plants were assigned an arbitrary score as 1 (very difficult to differentiate from BRRI dhan29), 2 (similar to BRRI dhan29 except for one or two traits), and 3 (different from BRRI dhan29 in more than two traits). In the BC_1_F_1_ generation, three individuals out of 428 plants were found to be highly similar to BRRI dhan29, while 230 plants were similar in phenotype. Out of 80 plants among four BC_2_ families (BC2-66, BC2-91, BC2-259, and BC2-280) in the BC_2_F_1_ generation, 64 individuals from three families (BC2-66, BC2-259, and BC2-280) were found to be similar to BRRI dhan29, while at BC_3_F_1_ generation, 45 plants comprising five families were similar to BRRI dhan29 in different phenotypic traits (Table 1).

#### Progeny Selection for Background Recovery

**FIGURE 3:**
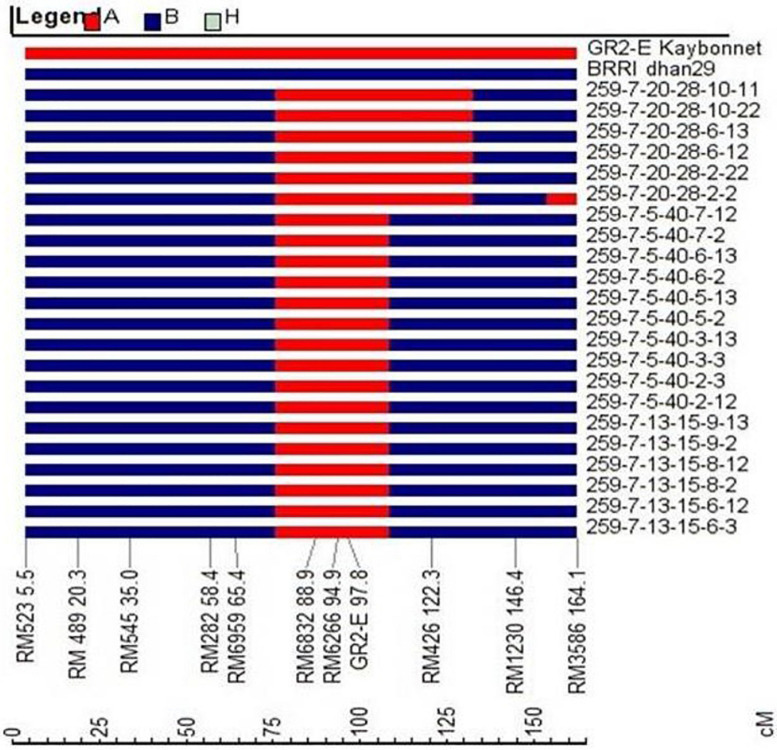
Graphical genotypes of 22 BC_3_F_4_ progenies showing introgression of donor segments on chromosome 3 (red color) containing GR2E locus from GR2E Golden Rice. Blue-colored segments signify the homozygous recurrent parent genome.

The plants with a hemizygous GR2E locus and phenotypically similar to BRRI dhan29 were used in background genotyping. Background selection was performed in two steps, firstly in the carrier chromosome (chromosome 3, where the GR2-E locus is located) with 9 SSR markers and secondly in the non-carrier chromosomes with an initial set of 86 SSRs. The SSR markers used in background genotyping were distributed over 12 chromosomes of rice at an average interval of 18.38 cM (6.1–43.7 cM). The recovery of the RPG in the carrier chromosome at BC_1_F_1_ generation among 233 plants ranged from 33.3 to 100%, while the overall recovery of RPG in all 12 chromosomes ranged from 58.9 to 84.2% (Supplementary Table S1). Three BC_1_F_1_ plants (Plant nos. 66, 259, and 280) showing maximum recovery of RPG (83.2–84.2%) were selected for the next cycle of backcrossing (Table 1). Sixty-three BC_2_F_1_ plants under three families (BC_2_-66, BC_2_-259, and BC_2_-280) and 45 BC_3_F_1_ plants under five families (BC3-259-6, BC3-259-7, BC3-259-10, BC3-259-11, and BC3-259-15) were used in background genotyping with up to 26 and 11 SSR markers (that were heterozygous in the previous backcross cycles) at BC_2_ and BC_3_ generations, respectively (Table 1). The overall recovery of RPG was 79.7–91.1% in the BC_2_F_1_ plants and 83.7–91.6% in the BC_3_F_1_ plants (Supplementary Table S1). Based on maximum recovery of RPG, 15 BC_2_F_1_ plants were used in backcrossing with BRRI dhan29 and 12 BC_3_F_1_ plants were selfed to produce BC_3_F_2_ seeds (Table 2). SSR genotyping was further performed on randomly selected two plants from each of 11 families tested at BC_3_F_4_ generation with 14 SSR markers that were heterozygous at BC_3_F_1_ generation along with newly identified 9 additional polymorphic SSR markers (to saturate the bigger gaps in the marker map) to quantify the genetic similarity/difference among the selected progenies. Results of these SSR analyses showed that all the 11 progenies were homozygous for the donor (GR2E) allele at two common loci, RM6832 and RM6266 on chromosome 3. Out of 23 SSRs analyzed in this generation, two loci were found fixed with donor alleles and six loci were found segregating among the test progenies (Table 3). All other loci were found fixed with recipient alleles (data not shown). All the progenies had homozygous donor alleles at more than two loci except BC_3_F_4_-259-7-13-15-8, in which both of the test plants were found fixed with donor allele for two loci (RM6832 and RM6266) on the long arm of chromosome 3 at 88.9 and 94.9 cM distance, respectively. It is important to note that RM6266 is located at 94.9 cM (∼23.78 Mb) on chromosome 3 and the transgene locus GR2E was integrated next to it at 24.51 Mb (Figure 3). The maximum RPG (98.1%) vis-à-vis minimum donor allele introgression (1.9%) was also in the two plants of BC_3_F_4_-259-7-13-15-8 (Table 3). In this generation, the test progenies reached a recovery of overall RPG ranging from 95.2 – 98.1%. These lines showed almost similar RPG recovery (95.2–96.8%) when analyzed with 314 polymorphic SNP markers with a relatively smaller proportion of donor allele introgression (Table 3). Background selection at BC_4_F_1_ and BC_5_F_1_ generations with 6K SNP chip showed more than 86 and 98% background recovery in the selected BRRI dhan29 GR2E lines.

**TABLE 2:** Results of different selection methods used in progeny selection at BC_2_F_1_ and BC_3_F_1_ generation of backcross introgression lines.

SL	BC_2_F_1_ cycle (*n* = 15)				BC_3_F_1_ cycle (*n* = 45)			
		
	Designation	Phenotypic similarity to recipient parent	%RPG	Total carotenoid content in self-fertilized seeds (μg/g)	Designation	Phenotypic similarity to recipient parent	%RPG	Total carotenoid content in self-fertilized seeds (μg/g)
1	BC_2_-66-2	2	88.6	3.5	BC_3_259-6-4	1	91.1	10.9
2	BC_2_-66-4	1	91.1	4.2	BC_3_259-6-26	1	88.9	12.8
3	BC_2_-66-8	2	89.2	2.9	BC_3_259-7-5	1	91.6	18.6
4	BC_2_-259-4	2	88.6	7.9	BC_3_259-7-7	1	87.9	18.4
5	BC_2_-259-5	2	82.4	5.3	BC_3_259-7-8	1	90.5	17.5
6	BC_2_-259-6	1	88.0	10.3	BC_3_259-7-12	1	90.0	16.7
7	BC_2_-259-7	1	87.3	7.5	BC_3_259-7-13	1	88.9	21.5
8	BC_2_-259-10	2	86.1	9.9	BC_3_259-7-20	1	89.5	19.2
9	BC_2_-259-11	2	81.6	9.6	BC_3_259-10-5	1.2	88.4	16.9
10	BC_2_-259-15	2	84.8	6.7	BC_3_259-10-14	1	89.5	14.6
11	BC_2_-280-6	2	89.9	3.6	BC_3_259-15-7	1	89.5	10.5
12	BC_2_-280-7	2	88.6	3.4	BC_3_259-15-15	1	86.3	10.0
13	BC_2_-280-19	2	90.5	2.6				
14	BC_2_-280-21	2	90.5	1.8				
15	BC_2_-280-35	2	88.6	2.4				

	Range (*n* = 15)			1.8–10.3				10.0-21.5
	Average	–	–	5.4		–	–	12.6
	S.E.	–	–	0.8		–	–	0.6

*RPG, recurrent parent genome; S.E., standard error.*

**TABLE 3:** Marker segregation in background selection of BC_3_F_4_ progenies of BRRI dhan29XGR2E population.

Designation	Markers								% of RPG and donor genome integression			
		
									SSR		SNP	
			
	RM6832	RM6266	RM426	RM3586	RM51	RM5704	RM 3701	RM19	Overall RPG (%)	% Donor allele	% BRRI dhan29 allele	% Donor allele
Chromosome	3	3	3	3	7	11	11	12				
cM	88.9	94.9	122.3	164.1	0.0	28.6	45.3	19.0				
BC_3_F_4_-259-7-13-15-6-3	A	A	B	B	B	H	A	B	96.6	2.9	95.7	4.3
BC_3_F_4_-259-7-13-15-6-12	A	A	B	B	B	H	A	B	96.6	2.9	95.9	4.1
BC_3_F_4_-259-7-13-15-8-2	A	A	B	B	B	B	B	B	98.1	1.9	96.0	4.0
BC_3_F_4_-259-7-13-15-8-12	A	A	B	B	B	B	B	B	98.1	1.9	96.2	3.8
BC_3_F_4_-259-7-13-15-9-2	A	A	B	B	A	B	B	B	97.1	2.9	95.0	5.0
BC_3_F_4_-259-7-13-15-9-13	A	A	B	B	A	H	H	B	96.2	2.9	94.2	5.8
BC_3_F_4_-259-7-5-40-2-12	A	A	B	B	B	A	A	A	95.2	4.8	92.5	7.5
BC_3_F_4_-259-7-5-40-2-3	A	A	B	B	B	A	A	A	95.2	4.8	92.5	7.5
BC_3_F_4_-259-7-5-40-3-3	A	A	B	B	B	A	A	A	95.2	4.8	92.5	7.5
BC_3_F_4_-259-7-5-40-3-13	A	A	B	B	B	A	A	A	95.2	4.8	92.6	7.4
BC_3_F_4_-259-7-5-40-5-2	A	A	B	B	B	A	A	A	95.2	4.8	92.7	7.3
BC_3_F_4_-259-7-5-40-5-13	A	A	B	B	B	A	A	A	95.2	4.8	92.6	7.4
BC_3_F_4_-259-7-5-40-6-2	A	A	B	B	B	A	A	A	95.2	4.8	92.4	7.6
BC_3_F_4_-259-7-5-40-6-13	A	A	B	B	B	A	A	A	95.2	4.8	92.5	7.5
BC_3_F_4_-259-7-5-40-7-2	A	A	B	B	B	A	A	A	95.2	4.8	92.5	7.5
BC_3_F_4_-259-7-5-40-7-12	A	A	B	B	B	A	A	A	95.2	4.8	92.7	7.3
BC_3_F_4_-259-7-20-28-2-2	A	A	A	A	A	B	B	B	95.2	4.8	96.6	3.4
BC_3_F_4_-259-7-20-28-2-22	A	A	A	B	A	B	B	B	96.2	3.8	96.8	3.2
BC_3_F_4_-259-7-20-28-6-12	A	A	A	B	B	B	B	B	97.1	2.9	96.0	4.0
BC_3_F_4_-259-7-20-28-6-13	A	A	A	B	B	B	B	B	97.1	2.9	95.9	4.1
BC_3_F_4_-259-7-20-28-10-22	A	A	A	B	A	B	B	B	96.2	3.8	96.8	3.2
BC_3_F_4_-259-7-20-28-10-11	A	A	A	B	A	B	B	B	96.2	3.8	96.6	3.4

#### Progeny Selection by Carotenoid Content

The carotenoids level in the GR2E Golden Rice was found to reduce over the storage in room temperature after harvest but it was stabilized after 6–8 weeks and remained almost static (unpublished data from a previous study conducted at IRRI in 2008). Thus, the carotenoid analysis was performed with selfed seeds of the BC_2_ and onward generations at 2 months of storage at room temperature after harvest. The TC content of polished grains of BC_2_F_2_ seeds of 15 plants at BC_2_ cycle (which were used in further backcrossing to produce BC_3_ seeds) ranged from 1.83 to 10.3 μg/g with an average value of 5.4 ± 0.8 μg/g. Maximum TC was observed for BC2-259-6 (10.3 μg/g) followed by BC2-259-10 (9.88 μg/g) and BC2-259-11 (9.57 μg/g). Five plants (BC2-259-6, BC2-259-7, BC2-259-10, BC2-259-11, and BC2-259-15) showing TC content of more than 6.5 μg/g, 81.6–88.0% RPG, and having a phenotype similar to BRRI dhan29 were selected from the BC2-259 family for further backcrossing with BRRI dhan29 (Table 2). In the BC_3_ cycle, TC content was analyzed from self-pollinated seeds (BC_3_F_2_) of 12 plants. TC content ranged from 10.0 to 21.5 μg/g with an average value of 12.6 ± 0.6 μg/g. The maximum TC was observed with BC3-259-7-13 (21.5 μg/g) followed by BC3-259-7-20 (19.2 μg/g), BC3-259-7-5 (18.6 μg/g), BC3-259-7-7 (18.4 μg/g), BC3-259-7-8 (17.5 μg/g), and thereafter decreasing. The causes behind the increased carotenoid content in this generation compared to its BC2 generation might be due to the post-transgenerational effect on carotenoid biosynthesis in rice seeds as reported by Datta et al. (2006). Datta et al. observed enhanced carotenoid levels in many T2 seeds when compared with their respective T1 seeds. A total of three BC_3_F_2_ progenies, BC3-259-7-5, BC3-259-7-13, and BC3-259-7-20 having TC 18.6, 21.5, and 19.2 μg/g, and RPG 91.6, 88.9, and 89.5%, respectively, were selected to advance the generation. In the BC_3_F_3_ generation, three homozygous lines for GR2-E locus were analyzed for carotenoids, and they showed 8.2 to 11.0 μg/g TC in the polished grain samples. Among 120 progenies from the BC_3_F_4_ generation, TC content ranged from 6.2 to 20.2 μg/g. Eleven progenies having TC > 8.0 μg/g were selected for evaluation of agronomic performance (Supplementary Table S2).

### Evaluation of Introgression Lines

#### Agronomic Evaluation Under SH Confinement

In the SH trial at IRRI, the BC_3_F_4_ NILs showed significant phenotypic variation among themselves for all the traits except the number of panicles/hill, panicle length, spikelets/panicle, grain yield/hill, milling yield, and head rice recovery (Supplementary Table S2). However, no significant difference between the test lines and the recurrent parent (BRRI dhan29) was observed in any trait except the number of panicles/hill (Table 4). All the NILs had significantly higher number of panicles/hill (7.2%) than that of BRRI dhan29. The SH trial at BRRI in 2015 with 170 BC_5_F_3_ lines did not show significant mean differences in yield from BRRI dhan29. The BC_5_F_3_ lines yielded an average of 11.9 g/hill with a range between 6.5 and 27.7 g and non-transgenic BRRI dhan29 yielded 14.6 g/hill. The results indicated that there were still many individual lines that yielded significantly higher than BRRI dhan29 (Supplementary Figure 2). In grain quality traits, the tested lines in both of the trials mentioned above did not show any significant mean difference from BRRI dhan29 except for amylose content. The average amylose content in the BC_3_F_5_ seeds harvested from the SH trial at IRRI in 2009 and in BC_5_F_4_ seeds from the SH trial at BRRI in 2015 was significantly lower than those in BRRI dhan29, although there was a wide range of variations in amylose content (16.2–29.4% with an average of 24.0 ± 2.4 μg/g) among the lines tested in the SH trial in 2015. TC levels in the BC_3_F_5_ seeds harvested from the SH trial in 2009 showed a range from 5.8 to 7.8 μg/g with an average value of 6.4 ± 0.56 μg/g, while it was 10.2 ± 0.23 μg/g in the BC_5_F_4_ seeds harvested from the SH trial in 2015 with a range from 5.3 to 19.4 μg/g (Table 4).

**TABLE 4:** Agronomic performance of BRRI dhan29 GR2E NILs in contained screen house and confined field trial conditions.

Parameter	SHT 2009 (*n* = 11)			SHT 2015 (*n* = 170)			CFT 2016 (*n* = 23)		
			
	BC_3_F_4_ NILs (avg ± *SD*)	BRRI dhan29	Mean difference	BC_5_F_3_ NILs (avg ± *SD*)	BRRI dhan29	Mean difference	BC_5_F_4_ NILs (avg ± *SD*)	BRRI dhan29	Mean difference
Days to maturity	126.4 ± 2.1	125.5	0.9	133.7 ± 2.6	128.5	5.2	147.4 ± 0.2	149.3	−1.9
Plant height	102.5 ± 6.8	104.1	−1.6	128.8 ± 4.9	132	−3.2	106.8 ± 0.4	107.9	−1.1
Panicles/hill	10.4 ± 0.8	9.7	1.0*	9.6 ± 1.1	10.3	−0.7	13.6 ± 1.1	12.9	0.7
Panicle length	26.6 ± 1.0	27.5	−0.9	26.6 ± 1.9	29.2	−2.6	26.6 ± 0.1	27.4	−0.8
Flag leaf length	40.9 ± 3.5	43.2	−2.3	–	–		–	–	
Flag leaf breadth	2.2 ± 0.1	2.2	∼0.0	–	–		–	–	
Spikelets/panicle	269.6 ± 60.5	257.7	11.9	173.9 ± 19.4	188.6	−14.7	191.7 ± 17.4	199.2	−7.5
% spikelet sterility	39.1 ± 13.6	31.2	7.9	58.3 ± 8.4	57.3	1.0	17.7 ± 0.5	21.5	−4.0
1000-grain weight	19.1 ± 0.7	19.9	−0.8	–	–		20.8 ± 0.9	21.4	−0.6
Grain yield/plant (g)	20.0 ± 5.4	23.6	−3.6	11.9 ± 4.3	14.6	−2.7	–	–	
Grain yield (t/ha)	–	–		–	–	–	7.0 ± 0.4	7.02	-0.02
Grain length (mm)	8.3 ± 0.1	8.5	−0.2	–	–	–	8.4 ± 0.02	8.6	−0.2
Grain breadth (mm)	2.4 ± 0.1	2.5	−0.1	–	–	–	1.8 ± 0.005	1.8	∼0.0
LB ratio	3.4 ± 0.1	3.6	−0.2	–	–	–	4.7 ± 0.15	4.8	-0.1
Milling yield (%)	77.5 ± 10.2	66.1	11.4	–	–	–	–	–	
Head rice yield (%)	54.9 ± 16.9	58.2	−3.3	–	–	–	–	–	
Amylose (%)	21.3 ± 0.4	30.3	−9.0**	24.0 ± 2.4	27.9	−3.9**	24.4 ± 1.8	28.0	−3.6**
Total carotenoids (μg/g)	6.42 ± 0.56	–	–	10.16 ± 0.23	–	–	10.99 ± 0.94	–	–
Range of TC (μg/g)	5.8–7.6	–	–	5.25–19.38	–		8.4–14.09	–	–

SHT, Screen house trial; CFT, confined field trial.

*,**Significant at 5 and 1% level of probability, respectively.

#### Agronomic Evaluation Under CFT

**FIGURE 4:**
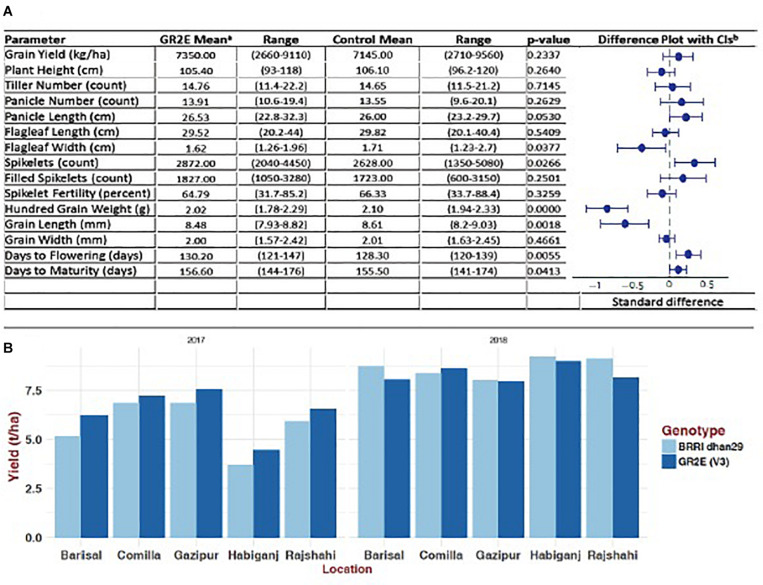
Agronomic performance of GR2E BRRI dhan29 Golden Rice compared to non-transgenic BRRI dhan29 control during 2017 and 2018 Boro season at five locations. **(A)** Difference plot with 95% confidence interval showing standardized differences of lead GR2E line from BRRI dhan29 in different agronomic traits: ^a^Values represent estimated marginal means (EMM) of three replicate measurements from the GR2E event and the control BRRI dhan29 plants grown at five locations in Bangladesh in the *Boro* seasons of 2017 and 2018 (*N* = 30). Data were subjected to linear mixed model analysis to generate EMM and estimates of statistical significance for any differences (*p* < 0:05); ^b^EMM calculated for each agronomic parameter for GR2E and control rice were compared in a meta-analysis using standardized differences (Heredia Díaz et al., 2017). When the 95% CI for the mean difference includes zero, the difference is not statistically significant at the 5% level (*p* < 0:05). **(B)** Comparative yield performance of the lead GR2E line and BRRI dhan29 in 2017 and 2018 Boro season.

The BC_5_F_4_ NILs of GR2E BRRI dhan29 showed no statistically significant differences from the non-transgenic BRRI dhan29 in any of the measured parameters except for amylose content, which was approximately 3.6% lower across the GR2E lines than for control BRRI dhan29 (Table 4). The transgenic NILs showed similar growth duration to the non-transgenic BRRI dhan29, although a few lines in individual plots showed significantly shorter days to maturity. No significant difference was observed in any other traits among the transgenic lines and between the transgenic lines and non-transgenic BRRI dhan29 except filled grains/panicle, spikelet sterility, and grain yield. Two NILs, IR 112060 GR 2-E:2-17-84 (#14, 18, 24) and IR 112064 GR 2-E:3-15-2 (# 8, 9, 33), had significantly higher number filled grains/panicle and significantly lower spikelet sterility than BRRI dhan29 and other NILs (Supplementary Table S3). The transgenic NILs yielded 6.2 to 7.7 t/ha with an average of 6.96 ± 0.38 t/ha, while the non-transgenic BRRI dhan29 yielded 7.0 t/ha. Amylose content among the entries was highly variable, ranging between 18.1 and 29.4%, while non-transgenic BRRI dhan29 had 28% amylose content. The average TCs analyzed from grains of individual plants of each entry showed a range from 8.4 to 14.09 μg/g with an average value of 11.99 ± 0.94 μg/g (Table 4). In multi-location trials conducted at five locations over two consecutive years, the tested lines did not show any significant difference of any measured traits from the non-transgenic BRRI dhan29 except grain width and flag leaf width, which was only 5% less on average across the locations (Figure 4). Across the locations, the NILs gave an average yield of 7.35 t/ha with a range from 2.66 to 9.11 t/ha, while the non-transgenic BRRI dhan29 yielded 7.14 t/ha on average. The transgenic NILs matured at 156.6 days over the locations with a range of 121–147 days and BRRI dhan29 matured at 155.5 days on average over the locations varying from141 to 174 days.

## Discussion

The GR2E event-specific STS marker was used in screening the lines with transgene in the advanced progenies derived from backcrossing. The transgene locus in a segregating backcross population is expected to be in its hemizygous form or absent. The presence of the GR2E locus in the backcross-derived progenies can be easily tracked by visual inspection of the grains (due to the yellow color imparted by the presence of carotenoids) after removal of the brown aleurone layer using a bleaching treatment. Thus, in this study, kernel color was considered in tracking the transgene within the progenies. However, the kernel color of naked seeds of F_1_ or BC_*n*_F1 was not visible due to the thick brown aleurone layer of the grains. Treatment of seeds with commercial bleach (2.5%) solution for 10 min was found effective to dissolve the aleurone layer of the grains (Figure 2) with no or minimal effect on viability (Biswas, 2011). In fact, genotyping costs for foreground selection were reduced at least by 50% following the phenotypic selection of seeds, as it enabled to discard all the seeds without the transgene (almost half of the seeds used in tracking transgene at each generation) before germination. The chi-squared test results of the individuals with yellow and white kernels were strongly in agreement with single-gene inheritance. These findings confirmed single copy transgene insert in GR2E as reported by Paine et al. (2005). Foreground selection with STS marker showed all hemizygous individuals grown from the seeds with yellow kernels as expected.

For progeny selection, maximum recovery of the RPG is usually considered (Neeraja et al., 2007; Septiningsih et al., 2009; Linh et al., 2012; Iftekharuddaula et al., 2015). However, full recovery of the background genome may not be always helpful to obtain progenies with better trait value of the introgressed trait. Iftekharuddaula et al. (2015) observed that *sub1* introgression lines having full recovery of the recipient genome were relatively poor in submergence tolerance compared to the recombinant lines that contained comparatively large homozygous donor segments. Similar results were also observed by Islam and Gregorio (2013) with the introgression of *SalTol* QTL into the BRRI dhan28 background. In the current study, some NILs with maximum RPG showed poor performance in carotenoid biosynthesis, vis-à-vis NILs with comparatively less RPG recovery showed better carotenoid productivity (Table 2). Therefore, carotenoid productivity level together with background recovery was considered for progeny selection at different backcross generations. Hence, any progeny having comparatively low TC values was not considered for further advancement, despite having higher background recovery. Five plants from the BC_2_F_1_ generation and three plants from the BC_3_F_1_ generation having TC values 6.7–10.3 and 18.6–21.5 μg/g, respectively, were selected for further advancement. The background recovery values of some of these selected plants were lower than other plants, but these NILs had comparatively higher TC values.

The introgression process usually completes in two to three cycles of backcrossing before fixation of the target locus. However, trait introgression together with complete or almost complete recovery of background sometimes takes additional backcross cycles, particularly when MABC is performed with *indica–japonica* crosses or crosses involving wild relatives. Earlier, Liu et al. (2016) performed six backcrosses to transfer the BPH resistance gene (Bph27t) from *indica* donor Ba into *japonica* cultivar NJ3. Similarly, Hirabayashi et al. (2010) required four backcross cycles to have RPG recovery 93.4–96% from crosses involving *Oryza rufipogon* L. The current study required five cycles of backcrossing to develop GR2E BRRI dhan29 NILs that showed no significant difference in any agronomic and grain quality traits except amylose content (24.4 ± 1.8%), which was on average 3.6% lower across the NILs than that in CFT harvest seeds of BRRI dhan29. This study completed the introgression process in two steps; BC_3_F_5_ NILs were developed first and evaluated for agronomic and grain quality traits and then selected lines were used in backcrossing further to develop advanced backcross generations of up to BC_5_ cycle. In fact, the initial target of this study was to finish MABC with three cycles of backcrossing. Although the top 11 BC_3_F_5_ NILs obtained 95.2–98.1% RPG with 104 SSRs and 92.5–96.8% RPG with 314 SNP, those NILs showed no significant mean difference from recurrent parent BRRI dhan29 for almost all of the agronomic and grain quality traits. On the other hand, these NILs had a TC content higher than the target level (8 μg/g). The NILs also showed significantly lower amylose content (21.3 ± 0.4%) than the non-transgenic BRRI dhan29 control (Table 4). This level of amylose content was below the acceptable limit (24%) of cultivar development in Bangladesh (Ministry of Agriculture, 2019; Swamy et al., 2019). The lower levels of amylose in the BC_3_F_5_ lines might be because of linkage drag from *japonica* donor genome donor source. The use of relatively lower marker resolution (average marker interval of 18.38 cM) at early backcross generations might be the cause of such linkage drag. The higher proportion of donor genome integration in these NILs also supports the linkage drag that was detected through comparatively higher-resolution genotyping with 314 SNP markers (Table 3). To increase the level of amylose content (at least 24%) in the NILs, two more cycles of backcrossing with BRRI dhan29 were performed and advanced to the BC_5_F_2_ stage. Background recovery at this stage was monitored with the 6K SNP-chip distributed over the genome. The recovery of the recurrent parent genome in the selected lines of BC_5_ generation was more than 98%.

**FIGURE 5:**
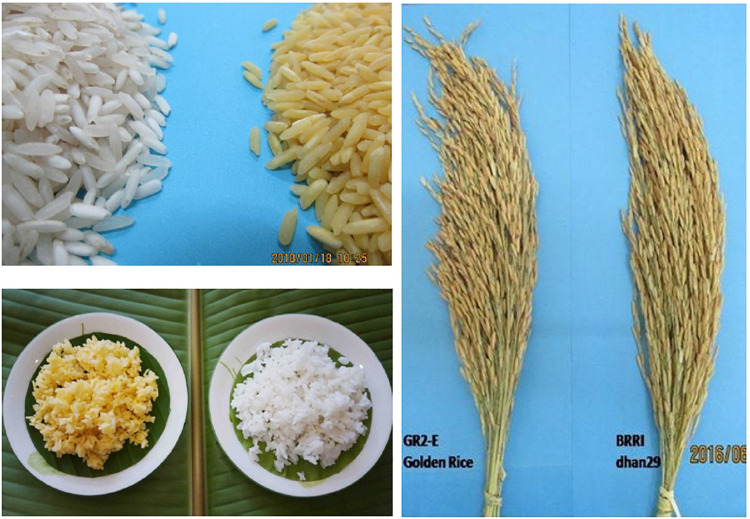
Panicle, uncooked, and cooked rice GR2E BRRI dhan29 and BRRI dhan29 (control) showing phenotypic similarity in grain shape and size except the yellow-orange color.

The BC_5_F_3_ NILs in the SH trial and their BC_5_F_4_ progenies in the CFT in Bangladesh displayed an almost identical phenotype to BRRI dhan29. Although the mean yield was not significantly different among the introgression lines, the individual lines showed considerable variation in amylose (22.6–26.2%) and carotenoid content (8.4–14.05 μg/g); thus, NILs selection was made considering amylose and TC content of individual lines that were either superior or almost similar to BRRI dhan29 in yield-related traits, growth duration, plant height, and grain quality. It is worthy to note that all these entries had an amylose content ranging from 22.4 to 27.9% with an average of 24.7% in BC_5_F_4_ seeds harvested from the SH trial in 2015. Patindol et al. (2014) reported that amylose content in the rice grain is greatly influenced by the growing environment and storage time. Eight NILs showing a cutoff value (24.5%) for amylose content at both BC_5_F_4_ and BC_5_F_5_ generations and having grain yield similar to BRRI dhan29 or higher were further advanced to test in multi-location sites to determine G × E on agronomic performance (Table 5). In the multi-location trials, no significant differences between GR2E BRRI dhan29 Golden Rice and non-transgenic BRRI dhan29 were observed, confirming that genetic modification did not have any unintended effect on plant growth, habit, general morphology, vegetative vigor, or grain yield of BRRI dhan29. Furthermore, the appearance of cooked and uncooked rice was similar to that of BRRI dhan29 except for yellow color, which indicated the presence of carotenoids (Figure 5). However, Bollinedi et al. (2017) reported a disruptive effect of the transgene locus from GR2-R1 Golden Rice on different agronomic traits in the genetic background of Swarna. In this report, Bollinedi et al. (2017) stated that an intragenic defect posed an unintended effect on agronomic traits manifesting reduced plant height, reduced panicle size, and incomplete panicle exertion, which in turn lowered yield in the recipient backgrounds at homozygous state of the transgene GR2-R1 locus. In the case of the GR2E event, no such alteration was observed in any agronomic traits of BRRI dhan29 and other backgrounds (Swamy et al., 2021). It is noteworthy that the GR2E event was found earlier as a defect-free event of Golden Rice (Oliva et al., 2020).

**TABLE 5:** Yield and grain quality attributes of selected BC_5_F_4_ NILs of GR2-E BRRI dhan29.

Designation	DM	PH	GL	GW	TGW	YLD	Amy	TC	GA
IR 112062 GR 2-E:14-40-7-8	147	112	8.4	1.8	21.8	7.2	25.3	10.9	Tr
IR 112062 GR 2-E:14-40-7-16	151	105	8.5	1.7	21.1	7.1	25.9	12.5	Tr
IR 112060 GR 2-E:2-9-89-16	144	108	8.4	1.8	20.4	7.5	25.9	11.2	Tr
IR 112060 GR 2-E:2-17-36-10	146	105	8.3	1.8	20.9	7.0	27.9	10.1	Tr
IR 112062 GR 2-E:14-40-7-21	145	112	8.4	1.8	20.2	7.8	24.5	10.7	Tr
IR 112060 GR 2-E:2-7-63-1	147	108	8.6	1.8	20.9	7.3	24.6	10.9	Tr
IR 112062 GR 2-E:14-40-7-23	143	103	8.5	1.8	20.8	7.4	25.8	9.8	Tr
IR 112060 GR 2-E:2-7-63-2	145	105	8.4	1.8	21.3	7.8	24.7	8.5	Tr
BRRI dhan29 (Ck)	148	108	8.8	1.8	21.4	7.0	28	–	Tr
LSD (0.05)	3.1	2.8	0.24	0.24	1.44	0.7	–	–	

*DM, Days to maturity; PH, Plant height (cm); GL, Grain length (mm); GB, Grain width (mm); TWG, Thousand grain weight (g); YLD, Yield (t/ha); Amy, Amylose (%); TC, Total carotenoids (μg/g) content at 2 months after harvest; Tr, Translucent; Ck, Check; LSD, Least significant difference.*

The carotenoid content in the selected transgenic lines varied from 8.5 to 12.5 μg/g with an average of 10.6 ± 1.16 μg/g when measured at 2 months after harvest. Considering the proportion of β-carotene (ca. 80%) in TC (Paine et al., 2005), a bioconversion factor of 3.8:1 (Tang et al., 2009) of Golden Rice β-carotene into vitamin A, a processing loss (milling and cooking) of ca. 40% (pessimistic assumption), the average intake of rice (Saltzman et al., 2013; Moura et al., 2016) by 1- to 3-year-old non-breastfed children (148 ± 5.8 g/day) or by non-pregnant non-lactating women (419 ± 8.4 g/day), and recommended daily intake (RDI) of vitamin A proposed by Institute of Medicine (2001) for children [300 μg retinol activity equivalent (RAE)] and women (700 μg RAE), GR2E BRRI dhan29 would be able to deliver approximately 66 and 80% of the RDI for children and women, respectively, assuming complete substitution of daily rice intake with Golden Rice. Based on yield performance and maturity duration over the locations, amylose, and TC contents, IR 112060 GR 2-E:2-7-63-2-96 was finally selected for further advancement.

## Conclusion

The transgenic NILs had recurrent parent genome up to more than 98% with genome-wide 6K SNP-chip set at BC_5_F_3_ generation and were almost identical to the non-transgenic BRRI dhan29 in SH and CFTs. The NILs also did not show any significant difference in almost all the morpho-agronomic and grain quality traits in multi-location trials at different agro-ecological zones under confined conditions. The TC contents of these lines were sufficiently enough to deliver up to 66 and 80% RDI of vitamin A for children and women, respectively. Therefore, considering yield, amylose content, and TC content, the best-selected NILs need further evaluation under open field conditions prior to deciding on commercial cultivation. Furthermore, the efficacy of the newly developed GR2E BRRI dhan29 Golden Rice in reducing VAD needs to be validated in a large malnourished community through a comprehensive feeding trial.

## Data Availability Statement

The original contributions presented in the study are included in the article/Supplementary Material, further inquiries can be directed to the corresponding author/s.

## Author Contributions

PB and BPMS conducted introgression and field testing. MAH initiated backcrossing. MAK conducted multi-location field testing. PB, BPMS, and RR conceived the idea. All authors reviewed and improved the manuscript.

## Conflict of Interest

The authors declare that the research was conducted in the absence of any commercial or financial relationships that could be construed as a potential conflict of interest.

## Supplementary Material

Supplementary Figure 1 Temperature and precipitation profile of the CFT sites during the growth phase of the crop in 2017.

Supplementary Figure 2 Yield performance of BC_3_F_4_ and BC_5_F_3_ NILs of GR2E BRRI dhan29 Golden Rice. Plate A represents the yield data from SHT 2009 and plate B for the yield data from SHT 2015.

Supplementary Table 1 The recovery of recurrent parental genome in the background selection at different backcross generations of GR2-E BRRI dhan29 Golden rice.

Supplementary Table 2 Agronomic and grain quality traits of the selected BC3F4 progenies of BR29XGR2-E in screenhouse at IRRI, 2010 DS.

Supplementary Table 3 Mean performance of GR2-E BRRI dhan29 under confined field trial at Boro season, 2016.
