# Supplementary figures and images for: Development and Field Evaluation of Near-Isogenic Lines of GR2-EBRRI dhan29 Golden Rice
Source: Front Plant Sci. 2021 Feb 25;12:619739. doi: 10.3389/fpls.2021.619739 (PMC7947304; doi:10.3389/fpls.2021.619739)

Supplementary Figure 1

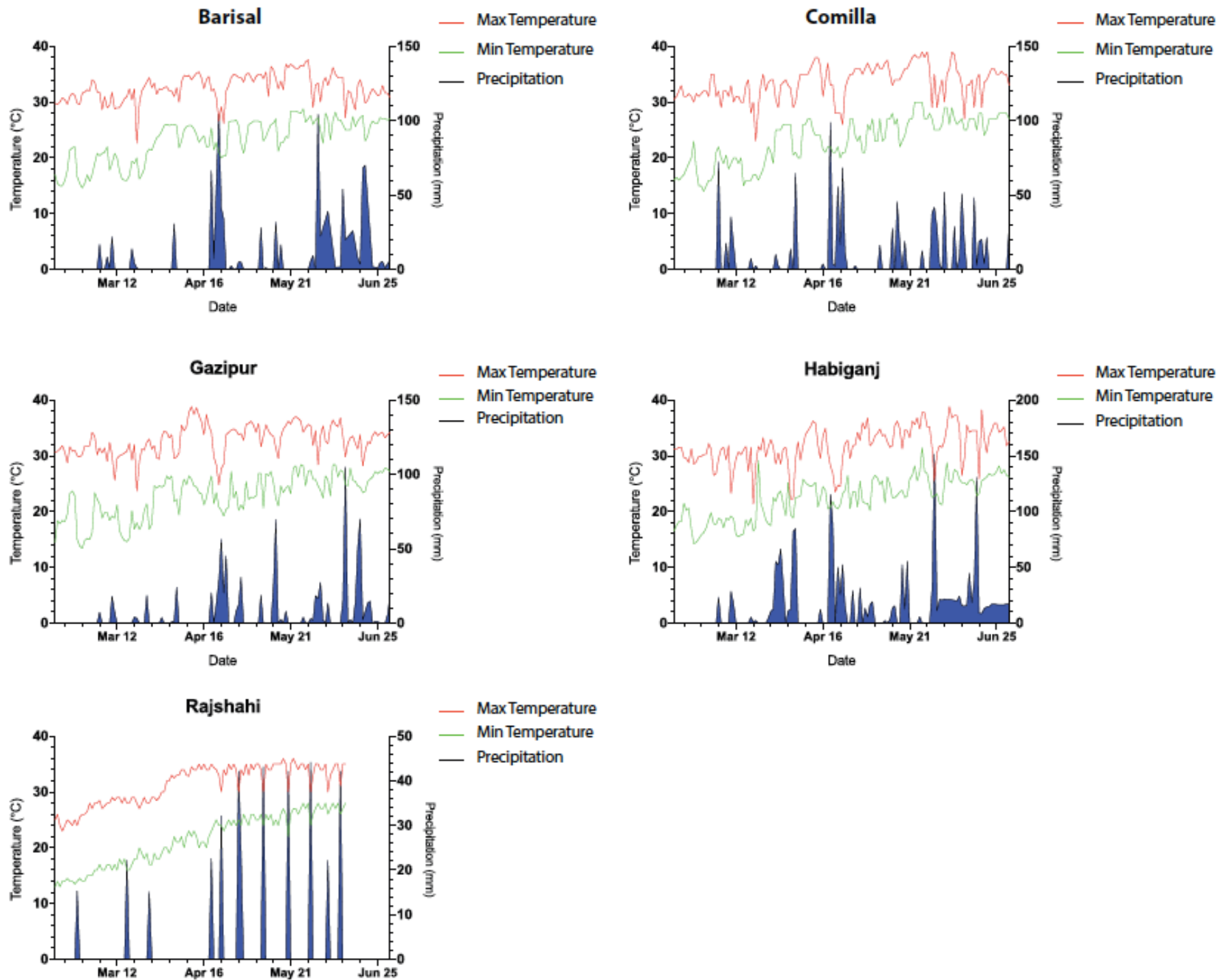

Supplement: Supplementary Figure 1 — Temperature and precipitation profile of the CFT sites during the growth phase of the crop in 2017. [file Data_Sheet_1.PDF]

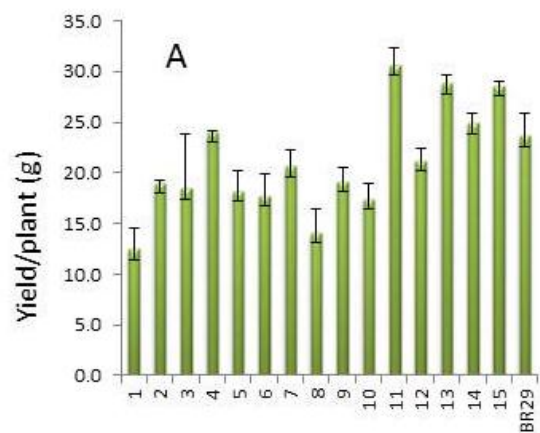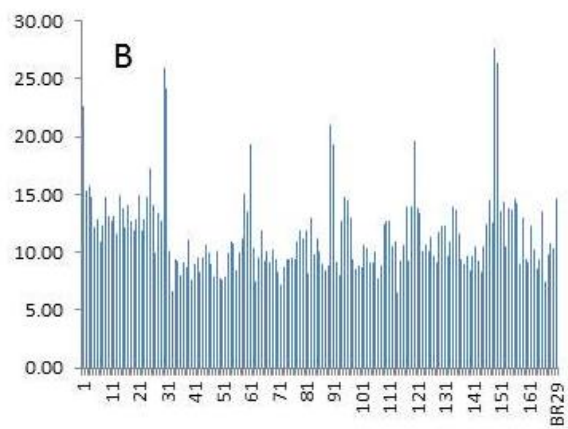

Supplement: Supplementary Figure 2 — Yield performance of BC3F4 and BC5F3 NILs of GR2E BRRI dhan29 Golden Rice. Plate A represents the yield data from SHT 2009 and plate B for the yield data from SHT 2015. [file Data_Sheet_2.PDF]
