# Supplementary material for: Development and Field Evaluation of Near-Isogenic Lines of GR2-EBRRI dhan29 Golden Rice
Source: Front Plant Sci. 2021 Feb 25;12:619739. doi: 10.3389/fpls.2021.619739 (PMC7947304; doi:10.3389/fpls.2021.619739)
Supplement: Supplementary Table 1 — The recovery of recurrent parental genome in the background selection at different backcross generations of GR2-E BRRI dhan29 Golden rice. [file Table_1.docx]

Supplementary Table S1: The recovery of recurrent parental genome in the background selection at different backcross generations of GR2-E BRRI dhan29 Golden rice

| Generation | Marker type* | Carrier chromosome | | All chromosome | |
| --- | --- | --- | --- | --- | --- |
|  |  | No. of plants used | % RPG | No. of plants used | % RPG |
| BC_1_F_1_ | SSR (95) | 233 | 33.3 -100 | 48 | 58.9 -84.2 |
| BC_2_F_1_ | SSR (95) | 63 (13+15+35) | 57.1 - 92.9 | 63 | 79.7 – 91.1 |
| BC_3_F_1_ | SSR (95) | 45 (8+11+9+4+13) | 92.9 - 100 | 45 | 83.7 -91.6 |
| BC_3_F_4_ | SSR (95) | - | - | 22 | 95.2 – 98.1 |
|  | SNP (384) | - | - | 22 | 92.5 – 96.8 |
| BC_4_F_1_ | SNP (6K) | - | - | 94 | >86% |
| BC_5_F_1_ | SNP (6K) | - | - | 94 | > 98% |

* Figures in the parenthesis are the number of markers used in background genotyping;

**Legend:**

Carrier chromosome, the chromosome that belongs GR2-E locus; RPG, Recurrent Parental Genome; SSR, Simple Sequence Repeat; SNP, Single Nucleotide Polymorphism; 6K, six thousand
