# Supplementary material for: Development and Field Evaluation of Near-Isogenic Lines of GR2-EBRRI dhan29 Golden Rice
Source: Front Plant Sci. 2021 Feb 25;12:619739. doi: 10.3389/fpls.2021.619739 (PMC7947304; doi:10.3389/fpls.2021.619739)
Supplement: Supplementary Table 2 — Agronomic and grain quality traits of the selected BC3F4 progenies of BR29XGR2-E in screenhouse at IRRI, 2010 DS. [file Table_2.docx]

Supplementary Table S2. Agronomic and grain quality traits of the selected BC_3_F_4_ progenies of BR29XGR2-E in screenhouse at IRRI, 2010 DS

| Genotype | Days to maturity | Plant height (cm) | Panicles/ plant (no.) | Panicle length (cm) | Flag leaf length (cm) | Flag leaf breadth (cm) | Flag leaf angle (°) | Spikelets/ panicle (no.) | % spikelet sterility | 1000g wt. (g) | Grain yield/plant (g) |
| --- | --- | --- | --- | --- | --- | --- | --- | --- | --- | --- | --- |
| BC_3_F_4_-259-7-13-15-6 | 123.0* | 93.2* | 10.5** | 26.4 | 44.5 | 2.25 | 22.0* | 272.6 | 47.7* | 18.7* | 19.0 |
| BC_3_F_4_-259-7-13-15-8 | 125.0 | 96.2 | 10.5** | 26.1 | 44.5 | 2.35* | 25.3 | 307.2 | 40.3 | 19.6 | 18.4 |
| BC_3_F_4_-259-7-13-15-9 | 127.0 | 97.7 | 10.5** | 26.5 | 42.8 | 2.25 | 28.6 | 305.7 | 29.2 | 19.5 | 24.0 |
| BC_3_F_4_-259-7-5-40-2 | 127.5 | 104.5 | 11.0** | 26.5 | 36.2* | 2.15 | 28.1 | 224.6 | 37.4 | 19.4 | 17.8 |
| BC_3_F_4_-259-7-5-40-3 | 127.5 | 103.5 | 11.0** | 26.6 | 39.5 | 2.20 | 30.5* | 222.9 | 34.8 | 19.6 | 20.7 |
| BC_3_F_4_-259-7-5-40-5 | 127.0 | 109.9 | 11.0** | 27.4 | 37.8* | 2.10 | 30.0 | 284.1 | 58.2** | 19.1 | 14.2* |
| BC_3_F_4_-259-7-5-40-6 | 127.0 | 110.0 | 10.5** | 26.9 | 36.8* | 2.05 | 29.8 | 289.4 | 50.6* | 18.8* | 19.2 |
| BC_3_F_4_-259-7-5-40-7 | 127.0 | 108.5 | 10.5** | 27.2 | 39.6 | 2.00 | 30.4 | 278.4 | 49.4* | 19.2 | 17.4 |
| BC_3_F_4_-259-7-20-28-2 | 126.5 | 106.2 | 9.5 | 26.7 | 38.5 | 2.05 | 28.5 | 266.9 | 29.7 | 18.7* | 21.2 |
| BC_3_F_4_-259-7-20-28-6 | 126.5 | 97.6 | 10.5** | 25.8 | 42.7 | 2.20 | 27.5 | 299.8 | 45.0 | 17.3** | 23.8 |
| BC_3_F_4_-259-7-20-28-10 | 127.0 | 107.2 | 10.5** | 27.3 | 44.2 | 2.30 | 26.4 | 278.3 | 30.6 | 18.5* | 23.6 |
| BR29 | 125.5 | 104.1 | 8.5 | 27.5 | 43.2 | 2.15 | 26.3 | 257.8 | 31.2 | 20.4 | 23.6 |
| Genotypic effect | 3.38* | 64.96* | 1.01 | 0.59 | 19.45* | 0.02* | 12.41* | 1548.2 | 188.4* | 1.67* | 19.56 |
| CV (%) | 0.73 | 4.14 | 5.9 | 3.92 | 5.87 | 4.02 | 6.64 | 10.34 | 17.14 | 3.34 | 18.91 |
| LSD (0.05) | 2.04 | 9.40 | 1.35 | 2.31 | 5.28 | 0.18 | 4.06 | 62.38 | 15.21 | 1.4 | 8.12 |
| LSD (0.01) | 2.88 | 13.16 | 1.90 | 3.26 | 7.44 | 0.26 | 5.73 | 88.03 | 21.47 | 1.98 | 11.88 |

* = significant at 5% level; ** = significant at 1% level

Supplementary Table S2. Agronomic and grain quality traits of the selected BC_3_F_4_ progenies of BR29XGR2-E in screenhouse at IRRI, 2010 DS (Contd)

| Genotype | Grain length (mm) | L:B ratio | Grain size and shape | Milling yield (%) | Head Rice (%) | % chalkiness | Amylose content (%) | TC (μg/g) at 2 MAH |
| --- | --- | --- | --- | --- | --- | --- | --- | --- |
| BC_3_F_4_-259-7-13-15-6 | 5.90 | 2.80 | Medium | 74.9 | 22.80* | 0 | 21.3** | 10.8 |
| BC_3_F_4_-259-7-13-15-8 | 5.80* | 2.60** | Medium | 73.6 | 34.85 | 0 | 20.5** | 9.6 |
| BC_3_F_4_-259-7-13-15-9 | 5.70** | 2.65** | Medium | 83.8* | 51.40 | 0 | 20.9** | 11.2 |
| BC_3_F_4_-259-7-5-40-2 | 5.95 | 2.80 | Medium | 74.2 | 42.90 | 0 | 22.1** | 11.9 |
| BC_3_F_4_-259-7-5-40-3 | 6.00 | 2.90 | Medium | 67.4 | 44.10 | 0 | 21.2** | 10.9 |
| BC_3_F_4_-259-7-5-40-5 | 6.00 | 2.95 | Medium | 74.2 | 48.65 | 0 | 21.7** | 20.2 |
| BC_3_F_4_-259-7-5-40-6 | 6.05 | 2.95 | Medium | 73.1 | 39.35 | 0 | 21.4** | 13.4 |
| BC_3_F_4_-259-7-5-40-7 | 5.90 | 2.85 | Medium | 72.5 | 55.70 | 0 | 21.4** | 10.3 |
| BC_3_F_4_-259-7-20-28-2 | 5.80** | 2.75* | Medium | 72.7 | 53.80 | 0 | 21.3** | 9.6 |
| BC_3_F_4_-259-7-20-28-6 | 5.75* | 2.70** | Medium | 73.8 | 59.15 | 0 | 21.2** | 10.0 |
| BC_3_F_4_-259-7-20-28-10 | 5.85 | 2.70** | Medium | 80.9* | 55.65 | 0 | 21.1** | 10.5 |
| BR29 | 6.05 | 2.90 | Medium | 66.1 | 53.15 | 0 | 28.3 | - |
| Genotypic effect | 0.028* | 0.028** | - | 46.68 | 221.96 | - | - | - |
| CV (%) | 1.75 | 2.28 | - | 7.85 | 22.12 | - | - | - |
| LSD at 5% level | 0.20 | 0.14 | - | 12.77 | 22.78 | - | - | - |
| LSD at 1% level | 0.29 | 0.20 | - | 18.03 | 32.14 | - | - | - |

* = significant at 5% level; ** = significant at 1% level; ns = non significant
