# Supplementary material for: Development and Field Evaluation of Near-Isogenic Lines of GR2-EBRRI dhan29 Golden Rice
Source: Front Plant Sci. 2021 Feb 25;12:619739. doi: 10.3389/fpls.2021.619739 (PMC7947304; doi:10.3389/fpls.2021.619739)
Supplement: Supplementary Table 3 — Mean performance of GR2-E BRRI dhan29 under confined field trial at Boro season, 2016. [file Table_3.DOCX]

Supplementary Table S3: Mean performance of GR2-E BRRI dhan29 under confined field trial at Boro season, 2016

| SL | Designation | DM | PH (cm) | PNH | PL (cm) | FGP | % St | GY (t/ha) | TGW (g) | GL (mm) | GW (mm) | AC (%) |
| --- | --- | --- | --- | --- | --- | --- | --- | --- | --- | --- | --- | --- |
| 1 | IR 112060 GR 2-E:2-11-18 (# 8, 22,28) | 145 | 107 | 14 | 26 | 150 | 21.5 | 7.4 | 20.5 | 8.4 | 1.79 | 23.6 |
| 2 | IR 112064 GR 2-E:3-6-99 (# 25, 26, 30) | 145 | 107 | 13 | 28 | 155 | 17.7 | 7.6 | 21.3 | 8.4 | 1.85 | 23.6 |
| 3 | IR 112064 GR 2-E:3-6-99 (# 4, 16, 18) | 148 | 109 | 14 | 25 | 152 | 18.7 | 7.2 | 22.1 | 8.2 | 1.88 | 23.2 |
| 4 | IR 112060 GR 2-E:2-9-16 (# 31, 34, 38) | 147 | 107 | 12 | 27 | 151 | 20.3 | 7.1 | 20.7 | 8.3 | 1.79 | 22.2 |
| 5 | IR 112064 GR 2-E:3-15-25 (#11, 29, 32) | 147 | 106 | 13 | 27 | 144 | 19.7 | 6.7 | 20.8 | 8.5 | 1.84 | 21.1 |
| 6 | IR 112060 GR 2-E:2-9-89(# 16, 23,25) | 146 | 105 | 13 | 27 | 157 | 22.2 | 7.3 | 20.4 | 8.4 | 1.83 | 24.8 |
| 7 | IR 112060 GR 2-E:2-9-89(#35, 36, 37) | 148 | 106 | 14 | 26 | 154 | 17.4 | 6.3 | 21.9 | 8.4 | 1.82 | 25.3 |
| 8 | IR 112060 GR 2-E:2-11-86 (#7, 9, 33) | 149 | 108 | 13 | 27 | 160 | 20.2 | 6.8 | 20.0 | 8.3 | 1.78 | 22.3 |
| 9 | IR 112064 GR 2-E:3-15-2 (# 8, 9, 33) | 147 | 107 | 14 | 26 | **182** | 14.4 | 6.6 | 20.7 | 8.3 | 1.81 | 24.4 |
| 10 | IR 112060 GR 2-E:2-7-63(# 12, 15, 18) | 150 | 106 | 13 | 28 | 150 | 22.0 | 6.7 | 20.4 | 8.4 | 1.80 | 25.6 |
| 11 | IR 112060 GR 2-E:2-7-63 (# 1, 2, 11) | 146 | 105 | 14 | 27 | 148 | 17.6 | 7.2 | 21.2 | 8.4 | 1.81 | 24.5 |
| 12 | IR 112060 GR 2-E:2-17-84 (# 12, 15, 17) | 145 | 107 | 14 | 26 | 160 | 14.1 | 7.0 | 20.4 | 8.7 | 1.79 | 22.5 |
| 13 | IR 112060 GR 2-E:2-17-84 (#14, 18, 24) | 146 | 105 | 13 | 26 | **192** | 11.4 | 7.3 | 21.2 | 8.5 | 1.81 | 21.3 |
| 14 | IR 112060 GR 2-E:2-17-84 (# 8, 27, 28) | 148 | 108 | 14 | 26 | 145 | 17.9 | 6.2 | 21.2 | 8.5 | 1.78 | 22.3 |
| 15 | IR 112074 GR 2-E:14-67-10 (# 25, 30, 31) | 146 | 108 | 15 | 27 | 176 | 13.4 | 6.9 | 20.0 | 8.3 | 1.82 | 23.9 |
| 16 | IR 112060 GR 2-E:2-17-36(# 9, 10, 14) | 148 | 108 | 14 | 26 | 144 | 20.9 | 6.5 | 21.3 | 8.4 | 1.80 | 24.3 |
| 17 | IR 112061 GR 2-E:12-9-94 (# 4, 27, 23) | 145 | 108 | 14 | 26 | 140 | 23.6 | 7.0 | 21.0 | 8.5 | 1.78 | 20.8 |
| 18 | IR 112062 GR 2-E:14-40-62 (# 2, 3, 24) | 146 | 107 | 12 | 27 | 144 | 19.3 | 6.8 | 20.9 | 8.6 | 1.85 | 21.5 |
| 19 | IR 112062 GR 2-E:14-40-7 (# 11, 20, 29) | 148 | 106 | 14 | 28 | 168 | 14.5 | 6.6 | 20.0 | 8.5 | 1.77 | 24.6 |
| 20 | IR 112062 GR 2-E:14-40-7 (# 12, 16, 19) | 147 | 107 | 14 | 27 | 158 | 17.8 | 6.9 | 21.1 | 8.5 | 1.74 | 24.8 |
| 21 | IR 112062 GR 2-E:14-40-7 (# 21, 25, 26) | 145 | 107 | 14 | 26 | 173 | 14.1 | 7.7 | 20.9 | 8.5 | 1.80 | 25.5 |
| 22 | IR 112062 GR 2-E:14-40-7 (# 8, 23, 24) | 145 | 105 | 14 | 27 | 157 | 13.1 | 7.1 | 20.6 | 8.5 | 1.79 | 24.5 |
| 23 | IR 112060 GR 2-E:2-11-62 (# 28, 26, 30) | 147 | 107 | 13 | 26 | 171 | 14.9 | 7.0 | 20.2 | 8.4 | 1.77 | 22.8 |
| 24 | BRRI dhan29 (Check) | 148 | 108 | 12 | 27 | 156 | 20.2 | 7.0 | 21.4 | 8.6 | 1.81 | 28.0 |
|  | LSD (0.05) | 3.09 | 2.82 | 1.70 | 1.60 | 22.03 | 5.30 | 0.70 | 1.44 | 0.24 | 0.24 |  |

Note: DM, Days to maturity; PH, Plant height; PNH, Panicle number per hill; PL, Panicle length; FGP, Filled grain per panicle; % St, percentage of sterility; GY, Grain yield, TGW, Thousand grain weight; GL, Grain length; GW, Grain width; AC, Amylose content.
